# Does non-invasive brain stimulation applied over the dorsolateral prefrontal cortex non-specifically influence mood and emotional processing in healthy individuals?

**DOI:** 10.3389/fncel.2015.00399

**Published:** 2015-10-14

**Authors:** Marine Mondino, François Thiffault, Shirley Fecteau

**Affiliations:** Centre Interdisciplinaire de Recherche en Réadaptation et en Intégration Sociale, Centre de Recherche de l'Institut Universitaire en Santé Mentale de Québec, Faculté de Médecine, Université LavalQuébec City, QC, Canada

**Keywords:** repetitive transcranial magnetic stimulation, rTMS, transcranial direct current stimulation, tDCS, mood, emotion, attentional processing, dorsolateral prefrontal cortex

## Abstract

The dorsolateral prefrontal cortex (DLPFC) is often targeted with non-invasive brain stimulation (NIBS) to modulate *in vivo* human behaviors. This brain region plays a key role in mood, emotional processing, and attentional processing of emotional information. In this article, we ask the question: when we target the DLPFC with NIBS, do we modulate these processes altogether, non-specifically, or can we modulate them selectively? We thus review articles investigating the effects of NIBS applied over the DLPFC on mood, emotional processing, and attentional processing of emotional stimuli in healthy subjects. We discuss that NIBS over the DLPFC can modulate emotional processing and attentional processing of emotional stimuli, without specifically influencing mood. Indeed, there seems to be a lack of evidence that NIBS over the DLPFC influences mood in healthy individuals. Finally, there appears to be a hemispheric lateralization: when applied over the left DLPFC, NIBS improved processing of positive stimuli and reduced selective attention for stimuli expressing anger, whereas when applied over the right DLPFC, it increased selective attention for stimuli expressing anger.

## Introduction

Non-invasive brain stimulation techniques (NIBS) such as repetitive transcranial magnetic stimulation (rTMS) and transcranial direct current stimulation (tDCS) can modulate human brain activity and connectivity (Shafi et al., 2012) and selectively improve or disrupt behaviors (Bikson and Rahman, 2013). They are increasingly used and one region that is often targeted with NIBS to modulate human behaviors *in vivo* is the dorsolateral prefrontal cortex (DLPFC).

Neuroimaging literature reports that the DLPFC plays a key role in mood (Davidson and Irwin, 1999), emotional processing (Herrington et al., 2005) and attentional processing of emotional information (Jacob et al., 2014). A meta-analysis however indicated that mood predominantly elicits activity in the medial inferior PFC, whereas attentional processing of emotional information mainly evokes activity in the DLPFC (Steele and Lawrie, 2004). Hemispherical specialization of emotional processing has also been proposed: activation in the left DLPFC has been associated with positive mood and processing positive stimuli, whereas activation in the right DLPFC has been linked to negative mood and processing negative stimuli (Canli et al., 1998).

Importantly, behavioral studies showed that mood, emotional processing, and attention can influence one another in healthy individuals. Experimentally induced depressed mood impaired identification of facial expressions and retrieving negative stimuli (Chepenik et al., 2007) and influenced rating of facial expressions as more negative (Bouhuys et al., 1995). Elevating mood improved implicit processing of happy faces (Quarto et al., 2014). Also, inducing negative attentional bias increased sadness (MacLeod et al., 2002).

Considering the importance of the DLPFC in mood, emotional processing, and attention and the interplay between these processes, when modulating its activity and connectivity with NIBS, one may wonder whether we are influencing these processes altogether non-specifically or whether we can modify them selectively depending on the targeted hemisphere and/or stimulation parameters. Here we aim at reviewing studies applying NIBS over the DLPFC of healthy subjects to modulate (1) mood, (2) emotional processing, and (3) attentional processing of emotional stimuli to decipher these in the interplay between these processes. Here, mood is defined as the emotional state. Emotional processing refers to processing the emotional content of stimuli (e.g., identification of facial expressions, perception of valence, and retrieval of emotional information). Attentional processing of emotional information refers to the attentional processes selecting and prioritizing relevant emotional stimuli.

## Can NIBS applied over the DLPFC modulate mood in healthy individuals?

Studies investigated whether NIBS applied over the DLPFC can modulate mood in healthy individuals (Table 1A). Pascual-Leone et al. (1996) reported that subjects rated higher anxiety and sadness levels and lower happiness level, as assessed with a 5-item visual analog scale (VAS), after receiving 10 Hz rTMS over the left DLPFC. Similarly, George et al. (1996) found that subjects rated lower happiness level after 5 Hz rTMS over the left DLPFC and lower sadness level after rTMS over the right DLPFC. Mood was assessed with a modified version of the NIMH mood scale. Schaller et al. (2011) found elevated mood after delivering nine sessions of 25 Hz rTMS over the left DLPFC when measured with the Beck Depression Inventory (BDI), but not with a 6-item VAS. Contrarily to these studies, others reported no significant mood change with high-frequency rTMS over the DLPFC. Mood assessed with 5-item VAS was not modulated with 20 Hz over the left DLPFC (Mosimann et al., 2000) or 10 Hz rTMS over the left or right DLPFC (De Raedt et al., 2010). Similarly, mood was not changed with 10 Hz over the left DLPFC as assessed with VAS and the Profile of Mood Scale (POMS) (Baeken et al., 2006) or 10 Hz over the right DLPFC as tested with the POMS (Baeken et al., 2010; Vanderhasselt et al., 2011), VAS or the Positive and Negative Affect Schedule (PANAS) (Baeken et al., 2008). Padberg et al. (2001) reported that 10 Hz rTMS over the left or right DLPFC had no impact on mood assessed by an 8-item VAS, however when targeting the left DLPFC subjects displayed increased frequency and shorter reaction times of laughing reactions when presented with funny stimuli. Hoy et al. (2010) combined 5 Hz rTMS over the left DLPFC with exposition of positive stimuli to modulate mood. This combination or either method alone (rTMS or exposition) had no impact on mood assessed by a 5-item VAS and an affective go-no-go task. Some studies used low-frequency rTMS to investigate mood. Mood was not influenced with 1 Hz over the left or right DLPFC as assessed with a 4-item VAS (Grisaru et al., 2001) or 0.6 Hz rTMS over the left or right DLPFC as tested with the POMS (d'Alfonso et al., 2000). Anxiety measured by the State-Trait Anxiety Inventory was reduced after subjects received 1 Hz rTMS over the right DLPFC (Schutter et al., 2001).

**Table 1:** **Summaries of the studies investigating the effects of non-invasive brain stimulation applied over the dorsolateral prefrontal cortex on mood, emotional processing, and attentional processing of emotional information in healthy individuals**.

**References**	**Study design**	**N (Males)**	**Mean age in years**	Site of stimulation^*^	NIBS parameters^**^	**Experimental outcomes (Time of assessment)**	**Main results**
**(A) EFFECTS OF NIBS ON MOOD**							
***rTMS studies***							
Schaller et al., 2011	Parallel Sham controlled 9 rTMS sessions	44 (44)	Range: 19–33	L DLPFC (5 cm anterior to M1)	25 Hz 15 trains of 2 s 8 s ITI 750 pulses Increasing MT across sessions (from 100 to 136.9%)	1. BDI 2. 6-item mood VAS: happy/unhappy, cheerful/sad, energetic/lack of energy, lively/gloomy, even-tempered/restless, serious/smiling (day 0, day 5, day 9)	Active vs. sham rTMS: 1. BDI: Reduced sum scores and scores on "libido", "fatigability" and "weight loss" at day 5 and 9 2. Mood VAS: No effect
Baeken et al., 2010	Parallel No sham	10 (0)	N/A	L DLPFC (MRI BN)	10 Hz 40 trains of 3.9 s 26.1 s ITI 1560 pulses 100% MT	1. POMS-32 (t0, t1)	Before vs. after active L DLPFC rTMS: 1. POMS-32: No effect Before vs. after active R DLPFC rTMS: 1. POMS-32: No effect
		10 (0)	N/A	R DLPFC (MRI BN)			
Hoy et al., 2010	Crossover Sham controlled rTMS combined with exposure to positive or neutral pictures	10 (4)	31.2	L DLPFC (10/20 EEG)	5 Hz 30 trains of 10 s 20 s ITI 1500 pulses 120% MT	1. AGN task with happy and sad words 2. 5-item mood VAS: sadness, happiness, tiredness, anxiety, pain-discomfort 3. Valence and arousal ratings on IAPS pictures (t0, t1)	Active vs. sham rTMS: 1. AGN task: No effect 2. Mood VAS: No effect 3. Valence and arousal ratings: No effect
Baeken et al., 2008	Crossover Sham controlled	27 (0)	25.2	R DLPFC (MRI BN)	10 Hz 40 trains of 4.9 s 26.1 s ITI 1560 pulses 110% MT	1.5-item mood VAS: sadness, tension, vigor, anger, tiredness 2. POMS-32 (t0, t1, t30)	Active R DLPFC vs. sham rTMS: 1. Mood VAS: No effect 2. POMS-32: No effect
	Crossover Sham controlled	20 (0)	25.6	L DLPFC (MRI BN)	10 Hz 40 trains of 4.9 s 26.1 s ITI 1560 pulses 110% MT	1.5-item mood VAS: sadness, tension, vigor, anger, tiredness 2. POMS-32 (t0, t1, t30)	Active L DLPFC vs. sham rTMS: 1. Mood VAS: No effect 2. POMS-32: No effect
Baeken et al., 2006	Crossover Sham controlled	28 (0)	28.7	L DLPFC (MRI BN)	10 Hz 40 trains of 3.9 s 26.1 s ITI 1560 pulses 110% MT	1.5-item mood VAS: sadness, tension, vigor, anger, tiredness 2. POMS-32 (t0, t1, t30)	Active vs. sham rTMS: 1. Mood VAS: No effect 2. POMS-32: No effect
Grisaru et al., 2001	Crossover Sham controlled	18 (7)	40.5	L DLPFC R DLPFC (5 cm anterior to M1 or M2)	1 Hz 1 single train 500 pulses 110% MT	1.4-item mood VAS: irritability, anxiety, depression, happiness (t0, t5, t10, t30, t240)	Active (either L or R DLPFC ) vs. sham rTMS: 1. Mood VAS: No effect
Padberg et al., 2001	Crossover No sham	9 (5)	29.8	L DLPFC R DLPFC (5 cm anterior to M1 or M2)	10 Hz 10 trains of 5 s 30 s ITI 500 pulses 110% MT	1.8-item mood VAS: mood, emotion, general state, anxiety, activity, physical condition, self-perception (t0, t1, t15) 2. Facial expressions recording with ultrasonic signal emitted by mouth and eyes muscles during a funny movie (t0, t1)	Active rTMS, L vs. R DLPFC: 1. Mood VAS: No effect 2. Facial expressions: Increased frequencies of laughing and shorter RT of laughing movements
Schutter et al., 2001	Crossover Sham controlled	12 (8)	28.4	R DLPFC (10/20 EEG)	1 Hz 1 single train 1200 pulses 130% MT	1. STAI 2. STAS (t0, t1, t35, t65)	Active vs. sham rTMS: 1. STAI: Reduced anxiety 2. STAS: No effect
Mosimann et al., 2000	Crossover Sham controlled	25 (25)	22.4	L PFC (5 cm anterior, 2 cm lateral to M1)	20 Hz 40 trains of 2 s 30 s ITI 1600 pulses 100% MT	1.5-item mood VAS: tiredness, happiness, sadness, pain, anxiety (t0, t20)	Active vs. sham rTMS: 1. Mood VAS: No effect
George et al., 1996	Crossover No sham	10 (6)	35	L DLPFC R DLPFC (5 cm anterior to M1 or M2)	5 Hz 10 trains of 10 s 1 s ITI 500 pulses 120% MT	1. NIMH mood scale 2. Forced-choice mood VAS 3. PANAS (t0, t30, t60, t90, t180, t480, t1440)	Active rTMS, L vs. R DLPFC: 1. NIMH mood scale: Reduced happiness and increased sadness 2. Forced-choice mood-VAS: No effect 3. PANAS: No effect Active rTMS, R vs. L DLPFC: 1. NIMH mood scale: Reduced sadness and increased happiness 2. Forced-choice mood-VAS: No effect 3. PANAS: No effect
Pascual-Leone et al., 1996	Crossover No sham	10 (4)	Range: 22–27	L DLPFC R DLPFC (5 cm anterior to M1 or M2) Mid PFC	10 Hz 10 trains of 10 s 25 s ITI 500 pulses 110% MT	1.5-item mood VAS: pain discomfort, sadness, happiness, anxiety, tiredness (t0, t1)	Active rTMS, L vs. R DLPFC: 1. Mood VAS: Decreased happiness and increased sadness Active rTMS, L vs. Mid PFC: 1. Mood VAS: Increased pain/discomfort, anxiety and sadness Active rTMS, R vs. L DLPFC: 1. Mood VAS: Increased happiness
***tDCS studies***							
Plewnia et al., 2015	Parallel Sham controlled	28 (28)	27.9	L DLPFC/R deltoid (10/20 EEG)	1 mA 20 min 35 cm^2^	1. PANAS 2. PASAT	1. PANAS: No effect on positive affect. Increase in “upset” item after sham vs. active tDCS 2. PASAT: Shorter inter-stimulus interval after anodal vs. sham tDCS Slower inter-stimulus interval were correlated to increased upset
Morgan et al., 2014	Crossover No sham	18 (9)	23.2	L DLPFC/R DLPFC R DLPFC/L DLPFC (10/20 EEG)	1 mA 12 min 9 cm^2^	1. PANAS 2. Motivational state questionnaire 3. Memory task with IAPS pictures (t0, t1)	Active tDCS, L DLPFC/R DLPFCvs. R DLPFC/L DLPFC: 1. PANAS: No effect 2. Motivational state questionnaire: No effect 3. Memory task: No effect
Motohashi et al., 2013	Crossover Sham controlled 4 tDCS sessions	12 (12)	22	L DLPFC/ supraorbital region (10/20 EEG)	1 mA 20 min 35 cm^2^	1. POMS-30 (day 0, day 4)	Active vs. sham tDCS: 1. POMS-30: No effect
Plazier et al., 2012	Crossover Sham controlled	17 (17)	21.5	R DLPFC/L DLPFC L DLPFC/R DLPFC O2/O1 O1/O2 (10/20 EEG)	1.5 mA 20 min 35 cm^2^	1. SUDS 2. POMS-32 3. PANAS 4. BISBAS (t0, t1)	Active (either four conditions) vs. sham tDCS: 1. SUDS: No effect 2. POMS-32: No effect 3. PANAS: No effect 4. BISBAS: No effect
**(B) EFFECTS OF NIBS ON EMOTIONAL PROCESSING**							
***rTMS studies***							
Balconi and Cobelli, 2015	Crossover Sham controlled	69 (31)	28.1	L DLPFC Pz (10/20 EEG)	5 Hz 90 trains of 1 s 5 s ITI 450 pulses 100% MT	1. Memory task with positive and negative words and pictures with high and low arousal (t0.5) 2. Valence and arousal questionnaire with words and pictures (t1)	Active rTMS, L DLPFC vs. Pz and sham: 1. Memory task: Increased accuracy and reduced RT for positive high arousal words and pictures 2. Valence and arousal questionnaire: No effect
Balconi and Ferrari, 2013	Crossover Sham controlled	27 (12)	Range: 21–36	L DLPFC Cz (10/20 EEG)	5 Hz 180 trains of 1 s 5 s ITI 900 pulses 100% MT	1. Memory task with positive and negative words among semantically related or unrelated distractors (t0.5)	Active rTMS, L DLPFC vs. Cz and sham: 1. Memory task: Reduced RT for positive targets and positive (related and unrelated) distractors in subjects with high and low anxiety level
Balconi and Ferrari, 2012b	Crossover Sham controlled	30 (13)	Range: 21–31	L DLPFC Cz (10/20 EEG)	5 Hz 90 trains of 1 s 5 s ITI 450 pulses 100% MT	1. Memory task with positive and negative words (t0.5)	Active rTMS, L DLPFC vs. Cz and sham: 1. Memory task: increased accuracy for positive vs. negative words in subjects with high and low anxiety level. Reduced RT for positive vs. negative words in subjects with high anxiety level
Balconi and Ferrari, 2012a	Crossover Sham controlled	27	Range: 21–37	L DLPFC Cz (10/20 EEG)	5 Hz 90 trains of 1 s 5 s ITI 450 pulses 100% MT	1. Memory task with positive and negative words among semantically related or unrelated distractors (t0.5)	Active rTMS, L DLPFC vs. Cz and sham: 1. Memory task: Reduced RT for positive vs. negative words and related vs. unrelated positive distractors
***tDCS studies***							
Conson et al., 2015	Crossover Sham controlled	16 (8)	Range: 22–30	L DLPFC/R DLPFC R DLPFC/L DLPFC (10/20 EEG)	1 mA 15 min 35 cm^2^	1. Recognition of facial expressions task	Active tDCS, R DLPFC/L DLPFCvs. L DLPFC/R DLPFCand sham: 1. Recognition of facial expressions task: Reduced RT for fearful faces in male but not female subjects
Nitsche et al., 2012	Crossover Sham controlled	14 (9)	33.3	L DLPFC/ supraorbital region Supraorbital region/L DLPFC (10/20 EEG)	1 mA 20 min 35 cm^2^	1.14-item mood VAS (t0, t15, t30, t45, t60, t120, t180, t240, t300, following morning)	Active tDCS, L DLPFC/supraorbital region vs. supraorbital region/L DLPFC and sham: 1. Mood VAS: No effect
	Crossover Sham controlled	17 (9)	24.9	L DLPFC / supraorbital region Supraorbital region/L DLPFC (10/20 EEG)	1 mA 10 min 35 cm^2^	1. Recognition of facial expressions task (t0, t0.5, t5, t10, t20, t30, t60)	Active L DLPFC /supraorbital region vs. supraorbital region/L DLPFC: 1. Recognition of facial expression task: Reduced RT for positive (t0.5–t10) and negative faces (t0.5) Active supraorbital region/L DLPFC vs. sham tDCS: 1. Recognition of facial expression task: Reduced RT for negative faces (t10–t20)
Peña-Gómez et al., 2011	Crossover Sham controlled	16 (0)	22.9	L DLPFC/M2 (10/20 EEG)	1 mA 20 min 35 cm^2^	1. Valence rating task with IAPS stimuli (t0.5) 2. Mood 5-item VAS: annoyance, contentment, hope, nervousness, sadness 3. PANAS 4. STAI-state (t0, t1)	Active vs. sham tDCS: 1. Valence rating task: Negative pictures were rated as less negative Change in valence rating negatively correlated to extraversion score 2. Mood VAS: No effect 3. PANAS: No effect 4. STAI-state: No effect
	Crossover Sham controlled	9 (0)	25.8	M2/L DLPFC (10/20 EEG)	1 mA 20 min 35 cm^2^	1. Valence rating task with IAPS stimuli (t0.5)	Active vs. sham tDCS: 1. Valence rating task: No effect
**(C) EFFECTS OF NIBS ON ATTENTIONAL PROCESSING OF EMOTIONAL INFORMATION**							
***rTMS studies***							
Vanderhasselt et al., 2011	Crossover Sham controlled	28 (0)	22.3	R DLPFC (MNI BN)	10 Hz 40 trains of 3.9 s 26.1 s ITI 1560 pulses 110% MT	1. Exogenous cueing task with neutral and angry faces (t0, t1) 2. POMS-32 (t0, t1, t30)	Active vs. sham rTMS: 1. Exogenous cueing task: Increased AB for angry faces 2. POMS-32: No effect
De Raedt et al., 2010	Crossover (*n* = 18) and parallel (*n* = 19) sham controlled	37 (0)	22.6	L DLPFC R DLPFC (MNI BN)	10 Hz 40 trains of 3.9 s 26.1 s ITI 1560 pulses 110% MT	1. Exogenous cueing task with neutral and angry faces during an fMRI scanning (t0, t30) 2. Mood 5-item VAS: sadness, tension, vigor, fatigue, anger (t0, t1, t40)	Active R DLPFC vs. sham rTMS: 1. Exogenous cueing task and fMRI: Larger disengagement score for angry faces associated with decreased activation in R DLPFC , dorsal ACC, and L SPG 2. Mood VAS: No effect Active L DLPFC vs. sham rTMS: 1. Lower engagement score for angry faces associated with increased activation in the L OFC, R DLPFC , dorsal/pregenual ACC, R SPG 2. Mood VAS: No effect
Leyman et al., 2009	Crossover Sham controlled	18 (0)	21.1	R DLPFC	10 Hz 40 trains of 3.9 s 26.1 s ITI 1560 pulses 110% MT	1. NAP task with happy, sad and neutral faces (t0, t1) 2. Mood 5-item VAS: sadness, tension, vigor, fatigue, anger (t0, t1, t40)	Active R DLPFC vs. sham rTMS: 1. NAP task: Decreased scores for negative faces 2. Mood VAS: No effect
	Crossover Sham controlled	22 (0)	24	L DLPFC (MNI BN)	10 Hz 40 trains of 3.9 s 26.1 s ITI 1560 pulses 110% MT	1. NAP task with happy, sad and neutral faces (t0, t1) 2. Mood 5-item VAS: sadness, tension, vigor, fatigue, anger (t0, t1, t40)	Active L DLPFC vs. sham rTMS: 1. NAP task: No effect 2. Mood VAS: No effect
Van Honk et al., 2002b	Crossover Sham controlled	8 (4)	Range: 20–26	R DLPFC (10/20 EEG)	1 Hz 1 single train 1200 pulses 130% MT	1. Emotional Stroop task with masked and unmasked neutral and fearful faces (t30)	Active vs. sham rTMS: 1. Emotional Stroop task: Decreased attention for unmasked fearful faces
van Honk et al., 2002a	Crossover with no sham	10 (0)	Range: 18–30	L DLPFC R DLPFC (5 cm anterior to M1 or M2)	0.6 Hz 1 single train 540 pulses 130% MT	1. Emotional Stroop task with neutral and angry faces (t1) 2. PEP (t0, t1)	Active rTMS, R vs. L DLPFC: 1. Emotional Stroop task: Increased attention for angry faces 2. PEP: Reduced PEP Correlation between increased attention and reduced PEP
d'Alfonso et al., 2000	Crossover No sham	10 (0)	Range: 18–30	L DLPFC R DLPFC (5 cm anterior to M1 or M2)	0.6 Hz 1 single train 540 pulses 130% MT	1. Emotional Stroop task with neutral and angry faces (t10) 2. POMS-32 (t0, t1)	Active rTMS, L vs. R DLPFC: 1. Emotional Stroop task: Decreased attention for angry faces 2. POMS-32: No effect Active rTMS, R vs. L DLPFC 1. Emotional Stroop task: Increased attention for angry faces 2. POMS-32: No effect
***tDCS studies***							
Wolkenstein et al., 2014	Crossover Sham controlled	28 (8)	30.9	R deltoid/L DLPFC (10/20 EEG)	1 mA 20 min 35 cm^2^	1. DWM (t0.5) 2. AIT with positive, neutral and negative pictures (t1) 3. PANAS (t0, t1)	Active vs. sham tDCS: 1. DWM: Reduced accuracy for negative vs. neutral and positive pictures 2. AIT: Longer RT for negative vs. neutral and positive pictures 3. PANAS: No effect
Clarke et al., 2014	Parallel Sham controlled “Attend threat” ABM + active tDCS “Avoid threat” ABM + active tDCS	17 (7) 20 (6)	19.6 19.6	L DLPFC/L superior trapezius (10/20 EEG)	1 mA mean 17 min 24 cm^2^	1. AB assessment task with neutral and threatening words (t0, t1)	“Attend threat” ABM combined with active tDCS vs. “attend threat” ABM combined with sham tDCS: 1. AB assessment task: Increased AB to threat “Avoid threat” ABM combined with active tDCS vs. “Avoid threat” combined with sham tDCS: 1. AB assessment task: Decreased AB to threat
	“Attend threat” ABM + sham tDCS	22 (7)	20.6				
	“Avoid threat” ABM + sham tDCS	18 (8)	19.9				
Feeser et al., 2014	Parallel sham controlled tDCS applied during emotional regulation (4 conditions: maintain neutral emotions, downregulate, upregulate, or maintain negative emotions)	42 (20)	28.5	R DLFPC/L supraorbital region (10/20 EEG)	1.5 mA 20 min Anodal: 35 cm^2^ Cathodal: 100 cm^2^	1. Arousal ratings on IAPS pictures (t0.5) 2. Skin conductance response (t0.5) 3. Gaze fixation (t0.5) 4. Multidimensional State Questionnaire (t0, t1)	Active vs. Sham tDCS: 1. Arousal ratings: Lower in the downregulation conditions. Higher and in the negative maintain in the upregulation condition 2. Skin conductance: Lower response in the downregulation condition. Higher response in the upregulation condition 3. Gaze fixation: No effect 4. Multidimensional State Questionnaire: No effect

* *Site of stimulation is provided as follows, for rTMS: coil position, for tDCS: anode/cathode position. The method used to define the target is provided as follows, (10/20 EEG), Electrode placements according to 10/20 EEG system; (MRI BN), Magnetic Resonance Imaging based neuronavigation*.

** *NIBS parameters are provided as follows, for rTMS: frequency, trains number and duration, ITI, number of pulses, intensity, for tDCS: intensity, duration, electrode size. AB, Attentional bias; ABM, Attentional bias modification task; ACC, Anterior cingulate cortex; AGN, Affective go-no-go; AIT, Arithmetic inhibition task; BDI, Beck depression inventory; BISBAS, Behavioral inhibition system and behavioral approach system; Cz, Central midline; DLPFC, Dorsolateral prefrontal cortex; DWM, Delayed response working memory task; IAPS, International affective picture system; ITI, Intertrain interval; L, Left; M1, Left primary motor cortex; M2, Right primary motor cortex; MT, Motor threshold; N, number of subjects; NAP, Negative affective priming; NIBS, Non-invasive brain stimulation; NIMH, National institute of mental health; OFC, Orbitofrontal cortex; O1, Left occipital cortex; O2, Right occipital cortex; PANAS, Positive affect and negative affect schedule; PASAT, Paced auditory serial addition task; PEP, Preejection period; POMS, Profile of mood states; Pz, Parietal midline; R, Right; RT, Reaction Time; rTMS, repetitive transcranial magnetic stimulation; SPG, Superior parietal gyrus; STAI, State-trait anxiety index; STAS, State-trait anger scale; SUDS, Subjective unit of distress schedule; t0, Baseline; t0.5, During stimulation, t1, Immediately after stimulation, tX, X minutes after stimulation; tDCS, transcranial Direct Current Stimulation; VAS, Visual analog scale*.

In regards to tDCS, anodal tDCS over the left dlPFC and cathodal over the right deltoid muscle suppressed upset induced by the Paced Auditory Serial Addition Task (Plewnia et al., 2015). There seems to be no other studies reporting significant changes in mood when targeting the DLPFC of healthy subjects. This has been tested with anodal over the left and cathodal over the right DLPFC or the reverse electrode montage (Plazier et al., 2012; Morgan et al., 2014), anodal over the left DLPFC and cathodal over the right supraorbital region or the reverse montage (Nitsche et al., 2012), anodal over the left DLPFC and cathodal over the primary motor cortex (M1) or the reverse electrode montage (Peña-Gómez et al., 2011), and anodal over the left DLPFC and cathodal over the right supraorbital region delivering four sessions (Motohashi et al., 2013).

In sum, most studies reported that NIBS does not significantly influence mood in healthy subjects. Those reporting positive findings indicated a hemispheric lateralization: targeting the left DLPFC induced both negative and positive mood, whereas targeting the right DLPFC elevated mood.

## Can NIBS applied over the DLPFC modulate emotional processing in healthy individuals?

Studies applied NIBS over the DLPFC of healthy individuals to investigate emotional processing, especially perception of valence, identification of facial expressions, and retrieval of emotional information (Table 1B). In regards to perception of valence, Peña-Gómez et al. (2011) found that negative stimuli were perceived as less negative after anodal tDCS over the left DLPFC and cathodal over the right M1. Furthermore, this effect was stronger in subjects with higher subclinical scores of introversion.

For identification of facial expressions, Nitsche et al. (2012) reported that subjects were faster at identifying faces expressing positive and negative emotions during anodal or cathodal tDCS over the left DLPFC, with greater effect during anodal tDCS and positive stimuli. Conson et al. (2015) found that healthy men, but not women, were faster at recognizing fearful faces after receiving anodal and cathodal tDCS over the right and left DLPFC, respectively.

For retrieval of emotional stimuli, healthy subjects were faster at recognizing positive stimuli (Balconi and Ferrari, 2012a,b, 2013), especially stimuli of high arousal (Balconi and Cobelli, 2015), after receiving 5 Hz rTMS over the left DLPFC. Morgan et al. (2014) observed no change on retrieval of emotional stimuli delivering anodal and cathodal tDCS over the left and right DLPFC, respectively, or with the reverse montage.

Overall, NIBS targeting the DLPFC, especially the left hemisphere, seems to modulate emotional processing in healthy individuals, such as perceiving negative stimuli as less negative, improving identification of positive stimuli, and enhancing retrieval of positive information.

## Can NIBS applied over the DLPFC modulate attentional processing of emotional information in healthy individuals?

Several studies tested the effects of NIBS over the DLPFC of healthy individuals on attentional processing of emotional information (Table 1C). Selective attention toward emotional information has been tested with high- and low-frequency rTMS over the right and left DLPFC. Attention to angry faces was increased when targeting the right DLPFC with 10 Hz rTMS (De Raedt et al., 2010; Vanderhasselt et al., 2011) and 0.6 Hz rTMS (d'Alfonso et al., 2000; van Honk et al., 2002a). Interestingly, increased attentional bias toward angry faces was positively correlated with subject's anxiety level (Vanderhasselt et al., 2011) and elevated sympathetic activity (van Honk et al., 2002a). Moreover, targeting the right DLPFC with 1 Hz rTMS reduced attention to fearful faces (Van Honk et al., 2002b). Attention to angry faces was also reduced with 0.6 Hz rTMS (d'Alfonso et al., 2000) and 10 Hz rTMS (De Raedt et al., 2010) when targeting the left DLPFC.

NIBS over the DLPFC has also been used to promote attentional training. Clarke et al. (2014) tested the effects of tDCS during two attention bias modification tasks: one task trains attention to attend threat, whereas the other trains attention to avoid threat. Subjects receiving tDCS with the anode over the left DLPFC and the cathode over the left superior trapezius muscle displayed increased attentional bias to threat when trained to attend threat, but decreased attentional bias to threat when trained to avoid threat.

For inhibitory control of emotional information, it has been shown that targeting the right, but not the left DLPFC with 10 Hz rTMS impaired inhibition of negative stimuli (e.g., reduced negative affective priming for negative stimuli; Leyman et al., 2009). tDCS with the cathode over the left DLPFC and the anode over the right deltoid muscle also impaired inhibitory control for negative but not positive or neutral stimuli (Wolkenstein et al., 2014).

tDCS with the anode over the right DLPFC and the cathode over the left supraorbital region facilitated cognitive reappraisal by increasing emotional responsiveness (arousal rating of negative picture and skin conductance response) during negative emotion upregulation or decreasing emotional responsiveness during negative emotion downregulation (Feeser et al., 2014).

Overall, NIBS seems to modulate attentional processing of emotional stimuli, and these effects seem to depend on the targeted hemisphere. Specifically, selective attention toward anger increased when targeting the right DLPFC, but decreased when targeting the left DLPFC. Also, NIBS over either the left or right DLPFC disrupted inhibitory control when processing negative but not positive or neutral stimuli.

## Discussion

We reviewed here studies investigating the effects of NIBS applied over the DLPFC on mood, emotional processing and attentional processing of emotional information in healthy individuals. Overall, NIBS can selectively modulate processing of emotional information without significantly influencing mood. Specifically, NIBS over the left DLPFC resulted in improving identification of positive facial expressions (Nitsche et al., 2012), rating negative stimuli as less negative (Peña-Gómez et al., 2011), and reducing attention toward anger (d'Alfonso et al., 2000; De Raedt et al., 2010), without influencing mood. NIBS over the right DLPFC increased attention toward anger without influencing mood (d'Alfonso et al., 2000; De Raedt et al., 2010; Vanderhasselt et al., 2011). NIBS applied over either the left or right DLPFC disrupted inhibitory control when processing negative stimuli without interfering with mood (Leyman et al., 2009; Wolkenstein et al., 2014). It remains unclear whether NIBS can significantly influence mood in healthy individuals as several studies reported negative findings (d'Alfonso et al., 2000; Mosimann et al., 2000; Grisaru et al., 2001; Padberg et al., 2001; Baeken et al., 2006, 2008; De Raedt et al., 2010; Hoy et al., 2010; Peña-Gómez et al., 2011; Vanderhasselt et al., 2011; Nitsche et al., 2012; Plazier et al., 2012; Motohashi et al., 2013; Morgan et al., 2014).

**Figure 1:**
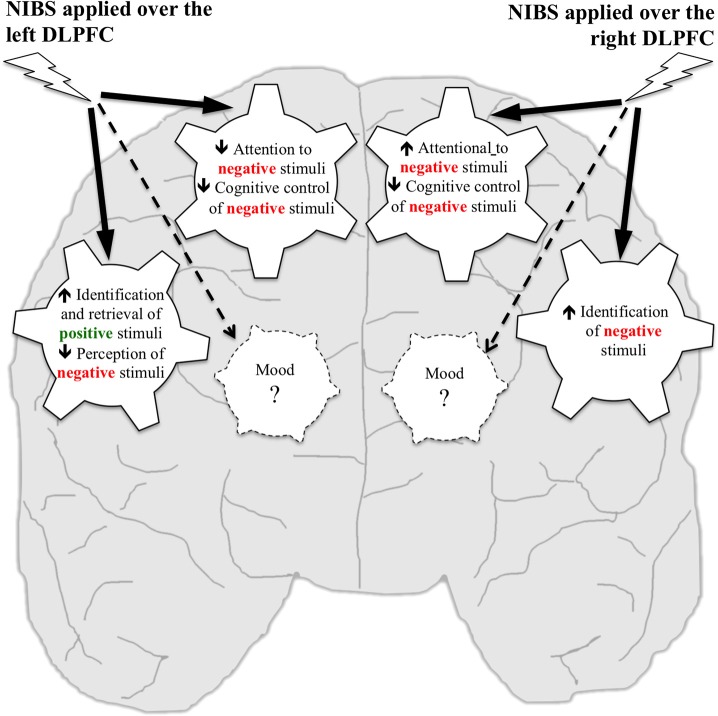
**Putative effects of NIBS over the left and right DLPFC on mood, emotional processing, and attentional processing of emotional information in healthy individuals**. Wheels represent processes that have been targeted using NIBS. Studies reported that NIBS applied over the left DLPFC increased identification and retrieval of positive stimuli, decreased perception of negative stimuli, decreased attention to negative stimuli, and cognitive control of negative stimuli, but no effect was reported on mood. NIBS applied over the right DLPFC increased identification of negative stimuli, increased attention to negative stimuli and decreased cognitive control of negative stimuli, but no effect was reported on mood.

We illustrate main findings in Figure 1. We propose that mood, emotional processing, attentional processing of emotional information are closely intertwined like wheels in a gear but that modulation of emotional processing or attention, as it has been induced with NIBS so far, may be insufficient to influence mood in healthy individuals. This is consistent with studies reporting that rTMS over the right DLPFC influenced processing of neutral stimuli such as intentional set switching (Vanderhasselt et al., 2006) and attention (Vanderhasselt et al., 2007) without impacting mood in healthy subjects. Similarly, one session of NIBS over the DLPFC reduced attentional bias for negative stimuli (Brunoni et al., 2014) and inhibitory control impairments independently from mood changes (Vanderhasselt et al., 2009a,b; Wolkenstein and Plewnia, 2013) in individuals with major depressive disorder (MDD). This is also in line with pharmacological work: administering a single dose of antidepressant medication to healthy subjects increased attention to positive words without changes in mood (Browning et al., 2007). Administering 7 days of antidepressant medication to healthy subjects reduced attention to fearful faces (Murphy et al., 2009), impaired identification of negative facial expressions and improved retrieval of positive stimuli (Harmer et al., 2004) without changes in mood.

Findings reviewed here support the hypothesis of hemispheric lateralization in processing emotional information. Neuroimaging studies showed that the left and right DLPFC are specialized in processing positive and negative emotions, respectively (Canli et al., 1998). As schematized in Figure 1, NIBS over the left DLPFC improved processing of positive stimuli and reduced attentional bias for negative stimuli, whereas NIBS over the right DLPFC improved identification of negative stimuli and increased attentional bias for negative stimuli.

In regards to stimulation parameters, it is not clear whether some are more effective than others to modulate mood, emotional processing or attentional processing of emotional stimuli in terms of rTMS frequencies (ranging from 0.6 to 25 Hz) or number of pulses (ranging from 450 to 1800 pulses). Higher intensity may induce greater effects: among the six studies using 100% of motor threshold (MT), four had positive results (all improved retrieval of emotional stimuli) and two had negative findings (no mood change), whereas the five studies using 130% of MT reported changes in mood and attention. Of note, Schaller et al. (2011) increased intensity from 100 to 130% of MT, along with the number of sessions, and reported no correlation between intensity and mood changes. For tDCS, anodal may induce greater effects than cathodal on emotional processing (Peña-Gómez et al., 2011; Nitsche et al., 2012), whereas it is not clear whether amplitude (ranging from 1 to 1.5 mA) or duration (ranging from 10 to 20 min) play an important role on these processes.

Some methodological considerations should be noted. First, mood, emotional processing, and attention to emotional information have been tested with various approaches and outcomes. For instance, mood has been assessed with self-rated homemade VAS on limited number of items (ranging from 4 to 14) to standardized questionnaires (POMS, PANAS), including clinical tools (BDI), whereas emotional processing and attention have been mainly measured in terms of accuracy (percent of correct answers) and response time (changes in milliseconds). These assessments and outcomes may not have the same sensitivity to capture NIBS-induced changes. As an example, Schaller et al. (2011) showed an effect on mood when assessed by the BDI but not by the 6-item VAS. The VAS (as well as the POMS and PANAS) require to rate mood on adjectives (e.g., delighted, timid) with no specific context, whereas the BDI consists of specific questions using contexts to assess mood. Second, NIBS-induced changes have been measured by comparing various NIBS conditions. Some found changes by comparing two active NIBS conditions (e.g., targeting the right vs. left DLPFC) and others used sham conditions that are considered as partially active (e.g., active rTMS with flipping the coil at a 45 or 90° angle away from the head, Loo et al., 2000). Third, the DLPFC has been located with several methods. For the rTMS studies, it has been located as the site that is 5 cm anteriorly from M1, anatomically defined with the international 10–20 EEG system (F3, F4) or with MRI and stimulated with a neuronavigation system. For the tDCS studies, the DLPFC has been located with the international 10–20 EEG system. Therefore, these methodological considerations may have contributed to the seemingly inconsistent results of the studies reviewed here, especially those studying mood in healthy individuals.

Only five studies out of 23 reported that NIBS over the DLPFC modulated mood in healthy subjects. This lack of clear evidence that NIBS can influence mood in healthy individuals differs from studies in individuals with treatment-resistant MDD. Applying high-frequency rTMS over the left DLPFC (O'Reardon et al., 2007), low-frequency over the right DLPFC (Fitzgerald et al., 2003), anodal and cathodal over the left and right DLPFC respectively (Brunoni et al., 2013) can reduce depressive symptoms in MDD. These differences on the effects of NIBS applied over the DLPFC on mood between healthy individuals and those with MDD may be explained by several factors. First, these populations differed in brain activity, especially within the DLPFC (Martinot et al., 1990), thus NIBS may modulate brain activity differently according to the studied populations. Moreover, studies in MDD delivered several NIBS sessions, whereas most studies in healthy subjects performed single NIBS sessions, which may be insufficient to induce significant mood changes. A meta-analysis analyzing the effects of tDCS in MDD reported a trend for greater mood improvement when more than 10 sessions were delivered as compared to a lesser number (Shiozawa et al., 2014). Here, two studies delivered repeated NIBS sessions in healthy subjects, one reported mood improvement after nine rTMS sessions (Schaller et al., 2011) and one found no change after four tDCS sessions (Motohashi et al., 2013).

The influence between mood and attention and between mood and cognitive control were not reported in the reviewed articles here focusing on healthy individuals. Interestingly, these findings differ from studies in individuals with MDD, which have suggested a close relationship between mood, emotional processing, and attention. It has even been hypothesized that one mechanism underlying elevated mood in individuals with MDD is that NIBS improve cognitive control and reduce attentional bias for negative stimuli (De Raedt et al., 2014).

To further characterize potential non-specific and selective effects of NIBS over the DLPFC on mood, emotional processing, and attention of emotional information, future studies should identify NIBS-induced neural changes linked to the observed behavioral changes. It is very likely that these effects are not limited to the DLPFC. For instance, De Raedt et al. (2010) showed that engagement and disengagement for angry faces implicated different neural networks. Engagement for angry faces induced activations in the left orbitofrontal cortex, right DLPFC, dorsal/pregenual anterior cingulate cortex (ACC), right superior parietal gyrus (SPG), whereas disengagement for angry faces elicited activations in the right DLPFC, dorsal ACC, and left SPG. Reducing depressive symptoms with rTMS in treatment-resistant MDD also appears to involve a complex neural network. Fox et al. (2012) proposed that targeted DLPFC sites leading to better clinical efficacy were negatively correlated with the subgenual cingulate. It would thus be interesting to investigate NIBS-induced neural changes that are overlapping or not when modulating mood, emotional processing and/or attention of emotional information in healthy individuals.

Another avenue for future investigation is brain state dependency, which is known to play an important role on the effects of NIBS (Lang et al., 2004; Silvanto et al., 2008). Only one study among those reviewed here likely primed the brain in a specific way. Hoy et al. (2010) presented their subjects with positive affective stimuli when delivering rTMS over the DLPFC. Results were however inconclusive as combination of exposition with rTMS did not modulate mood. Future work might consider guiding brain state when delivering stimulation. Personality traits also seem to influence the effects of NIBS over the DLPFC. NIBS seems to have greater effects in individuals with higher level of anxiety as compared to those with lower level of anxiety on retrieval of positive stimuli (Balconi and Ferrari, 2012b) and in individuals with higher levels of introversion on rating valence of negative stimuli (Peña-Gómez et al., 2011).

In sum, we cannot conclude whether NIBS over the DLPFC can selectively modulate one of these processes based on specific stimulation parameters and whether NIBS modulating a single process influences the others as only one or two of these processes have been studied within a same design. More studies measuring the effects of NIBS on these processes altogether are needed to test whether one can influence the others, or not. The underlying neurocognitive concepts of mood, emotional and attentional processes of emotional information still remain vague. Recent developments of NIBS, along with neuroimaging technics, should contribute to decipher these concepts. Our review highlights the potential for NIBS applied over the DLPFC to modulate emotional processing and attentional processing of emotional information in healthy individuals, whereas its effect on mood remains unclear.

### Conflict of interest statement

The authors declare that the research was conducted in the absence of any commercial or financial relationships that could be construed as a potential conflict of interest.
